# *Conceptual Scaffold Analysis* Using Mature Lay Concepts when Constructing Qualitative Theory

**DOI:** 10.1177/23333936261455156

**Published:** 2026-06-03

**Authors:** Janice M. Morse, Lauren Clark

**Affiliations:** 1 University of Utah, College of Nursing, Salt Lake City, Utah; 2 University of Alberta, Faculty of Nursing, Edmonton, Alberta; 3 Shapiro Family Endowed Chair in Developmental Disability Studies, School of Nursing, University of California Los Angeles

**Keywords:** scaffold, concept integration, qualitative research proposal preparation, theory development, qualitative mixed methods

## Abstract

An important part of a qualitative project is the proposal preparation required for ethics review and funding application, yet proposals are relatively undeveloped at the beginning of a qualitative study. Here we describe a new method, *Conceptual Scaffold Analysis,* for applying a relevant, mature lay concept (from the literature) as a focus within a research proposal. This method enables analyzing and integrating a concept into your proposed methods, expedites inquiry, provides an organizing framework, and reconstructs understanding. We present a selection of mature concepts pertinent to nursing that may be used for *Conceptual Scaffold Analysis.* As a complete method, *Conceptual Scaffold Analysis* may serve as the qualitative (QUAL) component in a mixed method project, enhancing the *qual* method for the sequential component, thereby enabling advanced theory development. We present an example of *Conceptual Scaffold Analysis* on a topic critical to nursing and public health: the importance of mothers’ trust in vaccinating their infants and children with developmental disabilities. Analysis *of trust* is used to organize the main QUAL component, contributing to a mixed methods design, as a scaffold for the analysis of the *qual* supplemental research study to follow. Thus, the concept analytic structure, developed from the scaffold analysis, expedites and enriches the subsequent qualitative component, strengthening an inductive mixed-method qualitative process.

In this article, we introduce a new technique, Conceptual Scaffold Analysis, for incorporating a mature lay concept to be used as a scaffold to identify, locate, and clarify the structure of data in a subsequent qualitative study. Because qualitative inquiry proposals, by definition, lack detail and precision, in some situations this problem interferes with funding and ethics board review. Conceptual Scaffold Analysis removes some of this methodological uncertainly by providing a conceptual analytic scheme that may be used as a framework for data analysis during proposal preparation, thereby enhancing a scheme for proposed data analysis, and/or used as the first component of a QUAL-*qual* mixed method design.

Mature concepts are those that have: a theoretical definition, preconditions, attributes, and outcomes identified.^1^ By identifying concepts that are already developed and described in the literature, conceptual scaffold analysis enables researchers to identify the attributes within concepts that are later used to deductively search, frame, and code data, and fill gaps or “soft spots” identified in the literature to produce a more robust concept applied to the topical area of interest.

Following a brief outline of the nature of conceptual structures, we describe how to develop an analytic scaffold from a relevant mature or mid-range concept that appears pertinent to an area of inquiry considered “soft”. The concept may have been identified with the topic but has not yet been explored in depth or has been only superficially associated with that phenomenon, setting, or population. Tentatively exploring the structural components of the concept and applying that structure to a new context expedites qualitative inquiry without threatening the inductive qualitative processes.

## Background

While it is used as a foundation prior to conducting a qualitative study, Conceptual Scaffold Analysis is not a conceptual framework *per se*. A conceptual framework used in quantitative inquiry may present a theory, identify and decontextualize variables, and suggest relationships among variables for hypothesis testing.

In qualitative inquiry, concepts occur at a higher level of abstraction. Mature concepts found in the literature have a conceptual structure with specified attributes defined. *Conceptual Scaffold Analysis* enables new interpretative insights to be identified, investigated, and confirmed; thus, unleashing the power of qualitative inquiry to reveal new findings during interpretive analysis. Using a relevant, mature concept as an analytic conceptual framework reveals “new knowledge” and “reconstructs understandings” (Corbin & Strauss, 2008, p. 22), builds theoretically dense findings that contribute to qualitative inquiry, and enables theory development in new areas at a higher level of abstraction.^2^ It is a same paradigm QUAL-*qual,* mixed methods design (Morse, 2017b; Morse & Neihaus, 2009).

## Method

To illustrate how concepts may be explored across contexts and incorporated into the qualitative proposal, we present *Conceptual Scaffold Analysis* as a new method for incorporating concepts into the initial proposal development phase of inquiry.

### Clarifying: What Is a Concept?

Concepts are abstractions of phenomena with common characteristics that have been identified over time and shared by members of a group or population, enabling communication. *Scientific concepts* describe empirical and fixed (rather than abstract) phenomena, have distinctive definitions, standardized characteristics linked to reality, and can be replicable across different situations. *Lay concepts* are more abstract without standard referents, but they allow meaning to be given to abstract ideas and those of most interest to qualitative researchers. Lay concepts are “embedded in reality” (Mitcham, 2017a, p. 95) and enable the elicitation of meaning, but may vary across conceptual domains, contexts, over time, and with usage. Understanding concepts is a window into participants’ perceptions, qualitative understanding, and meaning. It enables the focusing of qualitative data, the development of analysis, and finally the construction of innovative and useful theory.

Optimally, well-defined mature lay concepts are the best candidates for conceptual scaffold analysis, those that have been well-integrated yet widely used in everyday conversation and have been addressed and analyzed in the literature. We present a partial list of mature concepts potentially useful in *Conceptual Scaffold Analysis* (see List 1). To illustrate how conceptual scaffold analysis can streamline a proposed QUAL–>*qual* mixed methods study, in this article we selected the concept of *trust.* We used *Conceptual Scaffold Analysis* by applying trust in the context of mothers’ decision making for vaccine acceptance for their children with developmental disabilities, (see Clark, Morse et al., submitted). (Table 1)

**Table 1.** Partial List of Mature Concepts of Interest to Nursing

Concept	Reference
Becoming Resolute	Hall et al. (2009); In Morse^i^ (2017a), p 721-752
Caring	Morse, Solberg, et al., 1990; Morse, Bottorff, Neander, et al., 1991; Morse, Bottorff, et al., 1992; In Morse, 2017a), p. 282-292
Comfort/comforting	Morse (1992); Morse et al., 1994, p. 1995; Morse et al., 1995; Morse, Bottorff, Neander, et al., 1991; In Morse, 2017a), p. 293-313; 699-718.
Compathy	Morse, & Mitcham, In Morse, 2017a, pp. 201-289; 293; Morse, Mitcham, & van der Steen (1998);
Disembodiment (Pain)	Morse & Mitcham (1998); in Morse (2017a), pp. 220-218
Emotional Empathy	Morse, Anderson, et al., 1992; Morse, Bottorff, et al., 1992; in Morse (2017a), pp. 220-224
Enduring	Morse (2018), pp. 177-187; In Morse, 2017a), p. 465-469;
Ethical Sensitivity	Weaver & Morse (2006); Weaver, Morse et al. (2008); In Morse, 2017a), p. 389-409
Fatigue	Olson & Morse (2005), pp. 141-159.
Hope	Morse & Doberneck (1995); In Morse, 2017a), p. 254-264;
Nursing Presence	Finfgeld‐Connett, 2006; In Morse, 2017a, p. 575-583
Preserving self	Morse, Pooler, et al., 2014; In Morse, 2017a), p. 226-230
Resilience	Morse, Kent-Marvick et al., 2021.
Self-transcendence	Eldershaw & Morse, In Morse, 2017a, p. 357-380.
Social support	Hupcey (2007a), In Morse, 2017a, p. 333-344.
Suffering	Morse, Beres, et al., 2003; Morse (2005); In Morse, 2017a), pgs. 455-468; 653-659.
Trust	Hupcey (2002); Hupcey, Penrod, et al., 2001. In Morse, 2017a, pp. 344-348.
Uncertainty	Penrod (2007).

^i^References marked “In Morse, 2017a” refer to text in Morse, JM., *Analyzing and Conceptualizing the Theoretical Foundations of Nursing* (Springer).

### The Nature of Conceptual Structures

1. The Domain: The highest level of abstraction is the *domain* or the scope of the concept within a particular group or population. A domain is the scope limited by the *use* of the concept during interaction. For instance, a domain may be delineated by culture group, by linguistic group, by discipline, by occupation, by age groups within the population, by function (e.g., by sport or family,) and local nomenclatures (e.g., slang words or linguistic codes). Thus, a concept’s name may be broadly used within a group, but have a slightly different meaning even within the group. The ‘decoding’ of these meanings is a deductive strategy.

2. Attributes: The next analytic task, and at a lower level of abstraction, is the identification of concept *attributes,* or the main characteristics of concepts within the domain. *Attributes* are clusters of behaviors that have common characteristics and occur together. The are strongest at the center of the concept (and easiest to recognize) and weaken as they move towards the boundary (Morse, 2017a, pgs 106-107). Attributes do not have to be equally strong in all examples of the concept—they just have to be present. It is attributes that make a concept what *it is*, an example of a particular concept, and the same set of attributes occur in all instances of the concept. As attributes weaken, lose clarity, and grow fuzzier, the concept begins to merge with other concepts.

3. Attributes are not identical in every example of the concept. However (and this is the third level of “abstraction”), how these attributes manifest in each instance of the concept may not be uniform, with researcher interpretation resolving attribute fit for a particular context (Morse, 2017a, pp. 189-192). This involves an inductive stance—as the research proceeds, examples of usage and actions are sorted into categories and labeled as examples of attributes. Thus, at this stage, the researcher is working both inductively (while sifting and sorting) and deductively by placing the developing groupings of data into each identified attribute within the category name (a strategy called abduction [see Agar, 1996, 2021; Tavory & Timmermans, 2014]). And, as the examples accrue, so does the researcher’s level of certainty about the attributes of the concept.

But such analysis becomes more complex because psychosocial concepts are not “fixed” within the context. They change with usage, and they may be developed, maintained, or discarded over time by a group through everyday discourse. Once again, the examples of data collected from each group or population are not strictly equivalent. Still, they share similar characteristics or even a purpose that enables recognition that they belong to the class of attributes and are therefore a “fit”. Qualitative understanding/analysis of concepts is enhanced through *perceptive interpretation* of text or participant observation, so that model or theory building ensues beyond category formation. It provides a method of linking categories, and therefore providing access to hierarchical knowledge, or “generalization to other contexts”, or to change over time, using qualitative theory development methods.^3^

### Allied Concepts

To complicate matters, concepts emerge, have a “flip side”, mature, and even dissipate in variable processes. As concepts mature, they change form, develop attributes, and may be only partially related to the concept you are considering. Allied concepts may be considered as concepts associated with the main concept (i.e., containing some of the attributes but not all). Using the example of trust, faith and belief are allied concepts, perhaps even a precursor leading to or moving away from trust. Alternatively, faith and belief may be considered concepts in their own right, fulfilling a different purpose from the concept of trust.

## Scaffolding From Concepts Leads to Developing Qualitative Theory

“Scaffold” is a term for studying the structure of concepts (Morse, 2017a, pp. 137-138, 441-442) to enable the development of a conceptual domain. Of course, the “boundary” may change as the researcher’s understanding increases, but initially scaffolding prevents the researcher from feeling overwhelmed and “scattering” their analytic focus in the initial stages of inquiry.^4^ A scaffold offers the researcher a focus while maintaining an inductive stance that prevents forcing preconceived ideas onto the data. Again, this ‘boundary’—the scaffold—is not rigidly fixed, but provides a skeletal frame to facilitate data collection, and is particularly useful for descriptive comparison of two or more groups or populations.

### A Walkthrough Example Using Trust

For example, scaffolding the concept of trust enables the identification of a skeletal frame for the preliminary sorting of data into categories for analysis, as described in a research proposal. Studies employing *theoretically dense, rich descriptive methods* (e.g., phenomenology, discourse analysis, or a method using thematic analysis) as well as studies designed to *develop theory* (e.g., ethnography or grounded theory) benefit from conceptual scaffolding.

#### The Conceptual Scaffold Analysis Procedure

Analysis begins at the proposal phase of a project, when the researcher is steeped in the pertinent literature.

##### Identify the Main Concept

The first task is to identify the main *concept(s) associated with your particular topic* from the literature. At this point, the researcher must analyze the selected concept itself, but devoid of any particular setting or patient population.

Ask: Is the concept *mature*^5^ and has the concept been used in many contexts? Download significant articles, then analyze the literature that describes the concept *per se* to determine the attributes of the concept, and if these have been linked with their indicators in other studies. Using this literature, now examine how the concept has been used to reveal a particular perspective that may be applied when exploring your setting or patient population. Are conceptual attributes defined and applied across contexts? If so, the transference of this literature will enable you to describe the structure of the concept, its attributes, and even allied concepts that may be inherent in your setting and population, thereby sensitizing you and giving you a “leg up” as you start data collection and analysis.

If, in your literature review, the relevant primary concept is not immediately obvious, express the research question as a less confining problem statement or observation as you are searching within the literature of your proposed context. You may observe that your concept has been applied tangentially and does not appear prominently, but provided it is relevant, you may then move forward to explore that concept in your context, as described in the previous paragraph. It is important that the concept selected is reported in the literature relevant to the intended focus of the study, even if it is not well researched. This must be confirmed before you move into refining the problem statement. Finally, a targeted search of that *concept literature* must be conducted concurrently within the literature review for your topic, to assess what is actually known about the area from the perspective of your conceptual lens.

Thus, later, when you begin your study interviews with participants, the scaffold will help you identify and analyze your data and address your research problem/question. It makes your analytic process easier. Rather than searching for the concept and the attributes and even the allied (associated) concepts within your data, *Conceptual Scaffold Analysis* enables you to inductively “stand on the shoulders of giants”, expediting inquiry by using what is known (even though not in-depth) in the literature. Note that the structure of your proposed concept description is not a ‘conceptual framework’ designed to predict causal chains in a process, as used in quantitative inquiry, but rather an argument for the importance of your study—one that will provide conceptual grounding for your topic.

##### Analyze and Diagram the Concept

Within others’ published studies on similar topics you will find instances of conceptual attributes. Once your data collection/analysis commences, the attributes that you will identify as examples within your own interview data will be guided by the known attributes yet be specific to your context.

Using the example of *trust*, the four attributes of trust most frequently reported are: Uncertainty, Risk, Dependency, and Vulnerability (Cook, 2001; Heimer, 2001). As a qualitative researcher coding interview data, you may place similar data into categories inductively, label those categories, and then proceed to preliminarily match and compare your inductively identified categories with the known attributes identified in your conceptual scaffold. This is abductive reasoning, employing deductive thinking from the general to the specific process in the description of trust, while inductively building categories by identifying pertinent data.

In our proposed study of mothers’ trust in vaccines (see Clark, Morse, et al., submitted), we have selected two domains of trust pertinent for analysis that comprise this concept of trust in this context, as suggested by Heimer (2001): the Scientific Domain (including trust in the research, regulation of vaccines, pharmaceutical companies motives, and government-directed vaccination schedules for children) and the Interpersonal Domain (physicians, nurses, relatives who share opinions about vaccinations). Each of these two domains has different “actors”, recommendations, and perspectives. Taken together the Scientific and the Interpersonal domains of trust provide comprehensive—yet possibly conflicting—perspectives on parents’ indecision and lack of trust. At the conclusion of our vaccination study we intend to develop recommendations to enhance trust in vaccination by addressing Uncertainty, Risk, Dependency, and Vulnerability operating in the Scientific and Interpersonal Domains.

The selection of a domain varies with the researcher’s agenda, discipline, application of results (e.g., in health care [Gilson, 2006]), and even when trust is targeted to the general or particular (Schilke et al., 2021). This illustrates the versatility of scaffolding in planning your project.

**Figure 1.**
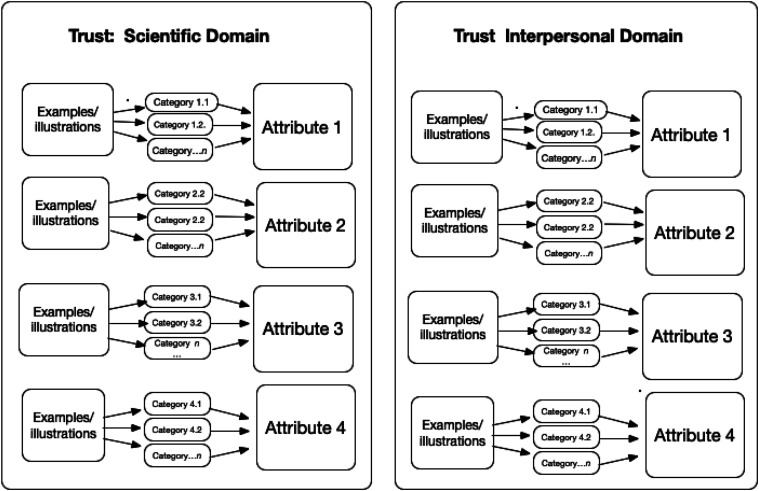
Schematic Representation of a Concept at the proposal stage and showing an abductive approach to the analysis of the literature

One important point is using the abductive process. At the proposal stage, after identifying the attributes, the investigator is working *deductively* by working from the literature to fill categories with examples and representative items. Those examples of conceptual attributes help generate questions to ask research participants to flesh out the attributes of the concept in context once the *qual* study commences. Then the investigator works *inductively* with their own data, sifting and sorting examples into categories, then into attributes. (This is the same process for each domain—data are kept separate). In the theory construction phase, those attributes are ordered as categories, with specified relationships within the domain (see Figure 1).

##### Prepare a “Straw Man” for Concept Data Collection

If the examples in the literature are rich, fascinating, relevant and pertinent to the proposed research context, then the investigator will content analyze those examples for attributes. It may even be possible to construct open-ended questions to ask participants that are rooted in the topics and categories demonstrated in the literature. The result will be an informed, balanced, and relevant set of interview questions based on attributes that can be used at the start of data collection.

From the four attributes of trust, research interview questions may be identified that target the participant’s perspectives, bringing the conceptual coding system to the level of the participant. For example, using the attribute of *Uncertainty,* one definition may be “unable to decide”. From this, the researcher may prepare interview questions to elicit specifics about uncertainty: “Is information sought?”, “Where?”, “By whom?”, “What type of information?”, and “Who is involved in making the decision?”. In this way the conceptual component is deductively organizing data collection and providing a scaffold for later analysis.

Based on this analysis, a draft interview guide may be prepared for the proposal, but to maximize its usefulness to your problem statement and research question, it may be modified once data collection commences and more information about the context becomes available. Furthermore, once data collection has commenced, the researcher must realize that working from example to category to attribute is now an ***inductive*** *process, a perceptual process using all the skills of qualitative interviewing and observation.* (See the example presented by Clark, Morse et al., submitted).

One possible mistake at this stage stems from naivete and appears as a failure to identify pertinent concepts from the literature. A second possible mistake is oversimplification that appears as a failure to untangle overlapping or multiple concepts embedded in the study context. These mistakes are understandable. Researchers are concerned about “leading the inquiry” and forcing results to fit extant theory. Mistakes of concept naïveté or surface understanding actually inhibit moving forward with the analysis, *as the concepts are usually the primary mechanism underlying what is happening in various contexts.* Using the concept literature and identifying conceptual attributes (i.e., the major characteristics of the concept) prepares the researcher to understand how the concept operates in a new research context. With data, the fit and misfitting of attributes illuminates how the processes of concept change actually *works*: how change occurs, and similarly, with that change, how concepts are modified. (Table 2)

**Table 2.** Comparison of 3 *future-oriented* Allied Concepts Compared With Trust

Attributes of the person	Trust	Hope	Faith	Belief
Goal Directed?	Present	Future oriented	Present and future	Future
Present state?	Uncertain	Untenable	Dissatisfied	Discomfort
​	Vulnerable	Compensated	Not vulnerable	Vulnerable
Involvement of others to achieve goal?	Dependent on others; risk to self; tests others	Self-identified goal	Goal-assisted Acceptance	Supported by others
Testing?	Incremental testing or comparison with expected outcome	Test self-resolve	Acceptance; No testing of the other	Acceptance; Not tested
Duration	Immediate and long term	Short or Long-term	Long-term	Variable
Level of Activity in Working towards goal	Active	Active	Passive	May be active (rapid) or (prolonged)

##### Consider the presence of allied concepts

Allied concepts may appear along with the major concept in the literature. Recall in our example allied concepts differ from each other and from trust in important ways. They have different attributes (are goal directed, future oriented); reflect different states; involve others and have different patterns of dependence; differ in duration; and vary in goal directedness. Three allied concepts appear alongside trust— hope, faith, and belief—yet these concepts can be differentiated from trust upon examination of attributes (see Table 1).

###### The Absence of the Concept: The Interpersonal Domain and Loss of Trust

Concepts have “two sides”, one that reveals the concept and the opposite “mirror image” side that does not support the main concept (Sztompka, 1999). Thus, the testing process in building trust may also have a negative outcome—that the trustee or authority figure fails the “test” and the trustor attempting to build trust loses any trust that has been gained.

This outcome must be considered. For instance, trust may be lost suddenly, erode over time, or operate temporarily until the trustor realizes there is a “problem” and, in turn, reacts to re-establish trust in the trustee. Placing oneself in a vulnerable position may even be ‘dangerous’ (Gilson, 2006) and the power differential between the trustee and trustor may even place the trustor in a position to be abused. Guardianship of vulnerable adults offers an example of the dangerous side of trust, with a powerless trustor experiencing financial or physical harm in the interpersonal domain if the trustee is untrustworthy. Some authors have noted there may be a process of negotiation to rebuild trust using probabilistic information (Senier, 2008), but usually, if trust is lost, mistrust within the trust or develops. If the trustor has the capacity to withdraw from the relationship, they will do so, later re-strategizing with other potential trustees. Questions may also be listed for consideration regarding the relationship between the attributes.

Questions Arising From the Scientific Domain
  • *Does vulnerability transition to uncertainty through risk assessment?*
  • *How is uncertainty managed? Vulnerability reduced?*
Consider
  • *What are the circumstances and mechanisms of losing trust?*
  • *If the trusting fails, what are the consequences for the trustor?*

##### Submit your proposal for ethics review

At this point your proposal is almost ready for submission to the ethics review board. Explain in your proposal that, as your research begins, you will locate participants and commence interviewing using an unstructured interview approach. As knowledge of your topic increases, your interview technique may change to a semi-structured interview schedule. Based on your developed content analysis in the QUAL phase, prepare semi-structured interview questions that will link your concept to your context.

## The Sequential (*Qual)*: The Theory Development Stage

The final aspect of the QUAL→*qual* study commences when the approving oversight committee, funding agency, and ethics review board sign off that your study is “worthy” of approval, marking the start of original data collection. Because your study depends on the concept development stage, it is important to publish both components together as a QUAL→*qual* design.

### Identifying Your Sequential Method for the Qual

Recall that developing a scaffold is a crucial method for focusing your study on a specific concept that has, so far, been poorly integrated into the literature on your topic of interest. With a conceptual scaffold, you avoid scrambling to locate and analyze relevant concepts on-the-fly once your data are obtained (Morse & Mitcham, 2002). You have a rich understanding of major (and perhaps allied) concepts, along with examples and categories applicable to the study setting. The next step is developing from this scaffold a useful, and hopefully profound and insightful, theory.

Using content analysis produces categories that may be analyzed descriptively or interpretively. Used alone, content analysis does not produce a very strong study (“What does it all *mean?)*.^6^ Once applied to a context, the content analysis produces data that can be sorted into categories developed from attributes. However, your study is structured to organize and begin conceptualizing your findings. This conceptualizing will be completed once you begin interviewing and come to understand more about your participants and the context. *Conceptual Scaffold Analysis* has sensitized you to significant categories suitable for data sorting and analysis—yet this is only one central step in analysis, not the final analysis.

Qualitative methods offer different strategies for describing and interpreting reality, and each provides a distinct perspective for approaching your data in line with your research agenda and purpose. The next step is to select your method, which will optimize your study, moving it from a superficial description to one that will make a solid contribution. Sort your methods into two types: 1) those that enhance thick, rich description and interpretation, and 2) those that facilitate abstraction and theoretical generalization.^7^

#### Enhancing thick descriptions and interpretation

Qualitative research does not end with the presentation of categories or themes. Rather, the concepts have provided you with a perspective that will be unique, and with additional conceptualization, result in significant in-depth results. This can be accomplished using the following methods: Phenomenology, conversational analysis, and narrative inquiry.

##### Phenomenology

Phenomenologists use anecdotes and interviews to get at the essence of meaning. Phenomenologists are sensitive to “descriptive, and interpretive, cognitive and evocative, propositional and poetic, analytical and synthesizing textual means” Van Manen (2014, p. 68).^8^

##### Conversational analysis

This is the analysis of the structure and process of social interaction. It includes analysis of the content of talk; interactional structures (turn taking), and methods of recording and assessing speech, in face-to-face interaction. Fine-grained analysis proceeds from video or audio recordings of actual interaction (Perakyla & Ruusuvuori, 2011).

##### Narrative inquiry

considers stories and their meaning as single units. Interview methods, such as narrative inquiry, begin with initial tenuous interviews, as the researcher actually learns to listen and be an excellent interviewer. The participant’s story is considered both the process of telling (the past) and a product (the story itself—living) (Clandinin, 2007). The role of the researcher is in the interpretation of the story and the emergent meaning as the story is lived. The theoretical product is usually described using a narrative format.

#### Facilitating abstraction and theoretical generalization

Two major methods fall under this heading: Ethnography (including participant observation) and grounded theory. This conceptualization will evolve through the interview process, as understanding of participants and context deepens, and interview questions are developed to gain an in-depth and specific understanding.

##### Ethnography

Ethnographic data collection includes strategies such as fieldwork, participant and non-participant observation, interviews, and review of relevant documents. Field research, including traditional anthropological research in a different culture, begins with a reciprocal process of the researcher and the community becoming used to each other (Pelto, 2013). To develop ethnographic findings theoretically, categories can be organized into relationships or hierarchy to address the research question.

##### Grounded theory

refers to several ‘sub-methods’ that are best used when exploring a process. The methods facilitate the development of categories, placing (ordering) the categories, identifying stages that facilitate the participant’s movement through the process, and pointing out transitions from one stage to the next. (See Corbin & Strauss, 2008; Glaser, 1978). Glaser (1978) recommends identifying a BSP (*Basic Social Process*) as the outcome of a grounded theory, and Corbin and Strauss (2008, p. 272) recommend identifying a *core category* to tie the theory together. The result is a generalizable midrange theory that fully explain the process. Grounded theories can be applied to other settings, and linked to other’s theories.

## Completion of Your Proposal?

Your qualitative proposal will not be specific and “tight.” You will not know how many interviews you will need, or how long the interviewing and observations may take. One way forward is to provide the ethics review board with regular progress reports. Explain the significance of successive specification of research processes as you gain experience in the field, offer links to various types of established theory (depending on the original purpose of your study) that informed the conceptual scaffold yet fall short of answering the research question, and demonstrate how you will conceptualize the results with hypothetical exemplars. Overall, the strength of the proposal will prevail as you craft an overview of how your findings will be compared with the concept as currently characterized in the literature.

## Last Words

*Conceptual Scaffold Analysis* benefits investigators by providing a jump-start at the proposal phase, focusing the study’s scope by aligning with concepts already elaborated in the literature. The conceptual attributes described in the literature are highly abstract, so examples of attributes vary across contexts, populations, and over historical time. Carl Mitcham reminds us that reality is not standardized or fixed. Reality is variable, flexible and changing: examples of conceptual attributes “come and go with fads and fancy. They develop, disappear, and change their names. And new ones are introduced” (Mitcham, 2017a, p. 96). Examples of attributes in every situation differ in detail. Attributes of a chosen concept that prove to be an excellent fit in one particular context and population may not be exactly the same in another context or situation. These modifications are extremely important. The abstractness of the concept makes it versatile, enables change, and facilitates insight (Morse & Mitcham, 2017a, pp. 95-96).

We weave the concept into the web of allied and other concepts, through pragmatic progress that links mature concepts to elaborations and specifications suited to new contexts, historical periods, and people. The classification of things “changes over time, as things themselves change” (Mitcham, p. 95). What counts is qualitative perception, insight, and detailed description concepts in context.

## Notes

1. To evaluate concept maturity, consider the concept’s coherence according to *epistemological criteria* (i.e., clear description of definitions, preconditions, attributes, boundaries and outcomes, and *pragmatic criteria* (i.e., how well the concept is operationalized, its usefulness to and fit with other phenomena of interest to the discipline), *linguistic criteria* (the consistency of the concept’s use across contexts) and *logical criteria* (i.e., how well the concept holds its boundaries through interaction with other concepts). See Morse, 2017a, pp. 392-393, also Chapter 11.

2. For an important example of this “uncovering” of knowledge, see Van Manen, Michael (2024).

3. This process is best described by Glaser (1978) throughout *Theoretical Sensitivity*.

4. The scaffold differs from Goffman’s frame analysis (Goffman, 1974), which is used for case-by-case analysis, and then comparisons between cases. A “scaffold” is simply an initial boundary and conceptual scheme for data collection.

5. Recall that a *mature concept* is clearly defined, its attributes labelled, their interrelationships described, and even the relationships of the attributes may be diagrammed. They will have been used in many different contexts and participant groups, which is a sign of their “generalizability” or applicability to other situations, indicative of their malleability and relevance (see, Morse, 2017a, pp. 155-156).

6. The content analysis places similar types together that occur together. Thematic analysis elicits examples from throughout the interview, and is therefore using a different method of sorting (see Blair, 2015).

7. An important Note: If your coding method is one that “enhances thick description” and the unit involves analysis by each participant individually, keep your codes from each participant separate. For ethnography and grounded theory, codes may be combined in categories. Good practice is to keep each participant’s number linked to each coded segment of text, so that, if necessary, you can return to the full transcript.

8. All major versions of grounded theory are discussed and compared in Morse, Bowers, et al., 2021.
